# Artificial intelligence in cancer target identification and drug discovery

**DOI:** 10.1038/s41392-022-00994-0

**Published:** 2022-05-10

**Authors:** Yujie You, Xin Lai, Yi Pan, Huiru Zheng, Julio Vera, Suran Liu, Senyi Deng, Le Zhang

**Affiliations:** 1 College of Computer Science, Sichuan University, Chengdu, 610065 China; 2 Laboratory of Systems Tumor Immunology, Friedrich-Alexander-Universität Erlangen-Nürnberg (FAU) and Universitätsklinikum Erlangen, Erlangen, 91052 Germany; 3 Faculty of Computer Science and Control Engineering, Shenzhen Institute of Advanced Technology, Chinese Academy of Sciences, Room D513, 1068 Xueyuan Avenue, Shenzhen University Town, Shenzhen, 518055 China; 4 School of Computing, Ulster University, Belfast, BT15 1ED UK; 5 Institute of Thoracic Oncology, Department of Thoracic Surgery, West China Hospital, Sichuan University, Chengdu, 610065 China; 6 Key Laboratory of Systems Biology, Hangzhou Institute for Advanced Study, University of Chinese Academy of Sciences, Chinese Academy of Sciences, Hangzhou, 310024 China; 7 Key Laboratory of Systems Health Science of Zhejiang Province, Hangzhou Institute for Advanced Study, University of Chinese Academy of Sciences, Hangzhou, 310024 China

**Keywords:** Systems biology, Tumour biomarkers, Cancer

## Abstract

Artificial intelligence is an advanced method to identify novel anticancer targets and discover novel drugs from biology networks because the networks can effectively preserve and quantify the interaction between components of cell systems underlying human diseases such as cancer. Here, we review and discuss how to employ artificial intelligence approaches to identify novel anticancer targets and discover drugs. First, we describe the scope of artificial intelligence biology analysis for novel anticancer target investigations. Second, we review and discuss the basic principles and theory of commonly used network-based and machine learning-based artificial intelligence algorithms. Finally, we showcase the applications of artificial intelligence approaches in cancer target identification and drug discovery. Taken together, the artificial intelligence models have provided us with a quantitative framework to study the relationship between network characteristics and cancer, thereby leading to the identification of potential anticancer targets and the discovery of novel drug candidates.

## Introduction

As one of the cutting-edge cancer treatments, targeted drug therapy has the advantages of high efficiency, few side effects, and low drug resistance for patients^1^. However, there are several drawbacks to the existing targeted therapies, such as a few druggable targets^2^, ineffective coverage of the patient population, and the lack of alternative responses to drug resistance in patients^1^. Therefore, identifying novel therapeutic targets and evaluating their druggability^3,4^ becomes the current cancer research focus of targeted drug therapy.

Since we have difficulty in comprehensively understanding the pathogenesis of cancer due to the complexity of the disease^5^, most of the current targeted drugs are developed based on the experimentally validated hypothesis that can explain a possible mechanism underlying carcinogenesis but ignore other facts of the disease^6^. As a result, these therapies could have undesired impacts on normal tissues and even provoke serious side effects for patients^7,8^.

To elucidate the molecular mechanisms underlying cancer genesis, interactome data can be comprised and modelled in network structures in which components are biological entities (e.g., genes, proteins, mRNAs, and metabolites) and edges are associations/interactions between them (e.g., gene co-expression, signalling transduction, gene regulation, and physical interaction between proteins^9–14^). Artificial intelligence biology analysis algorithms are effective method to process the biological network data, which build machines or programs to simulate human intelligence, so as to implement classification, clustering and prediction tasks in biological network^15^. Therefore, artificial intelligence algorithms can effectively tackle the complexity of cancer that arises from interactions between genes and their products^16,17^ in biological network structures, so as to improve our understanding of carcinogenesis^11,12,18–22^ and explore novel anticancer targets^23–29^.

**Fig. 1:**
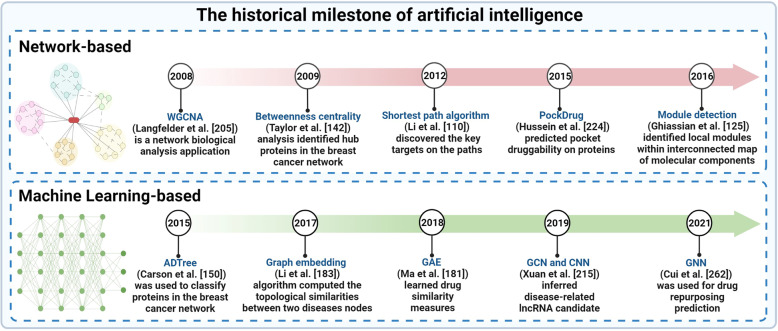
The historical milestones of network-based and ML-based biology analysis. (Created with BioRender.com)

Over the past few decades, we have seen a fast development of artificial intelligence biology analysis algorithms. To make this study easy to understand, we not only divide these artificial intelligence algorithms into network-based biology analysis algorithm and machine learning-based (ML-based) biology analysis algorithm according to the data of biological network structure, but also employ Fig. 1 to describe the historical milestone for these artificial intelligence biology analysis algorithms.

On the one hand, network-based biology analysis algorithms provide a variety of alternative network approaches to identify cancer targets. More importantly, various network-based biology analysis algorithms can investigate network data from different perspectives, therefore they can compensate each other to provide accurate biological explanations^30^.

On the other hand, ML-based biology analysis^31–33^ not only can efficiently handle high throughput, heterogeneous, and complex molecular data, but also can mine the feature or relationship in the biological networks. Thus, we should develop more ML-based biology analysis algorithms to provide such advanced biology analyses that can allow precise target identification and drug discovery for cancer.

Although artificial intelligence biology analysis has been widely used to improve our understanding of carcinogenesis, to the best of our knowledge, there is no systematic review that introduces the scope of related research and explains the network-based and the ML-based biology analysis algorithms to identify novel anticancer targets and discover drugs. Therefore, in the next section, we will describe the scope of artificial intelligence biology analysis for novel anticancer targets investigation. In the third section, we will introduce the basic principles and theory of commonly used artificial intelligence biology analysis algorithms. Then, we will briefly review and discuss studies that utilize network-based and ML-based biology analysis for cancer target identification and drug discovery. Finally, we will summarize the content of the article, discuss the limitations and challenges faced by the community, and point out the potential of artificial intelligence biology analysis to identify the therapeutic targets and discover drugs for cancer.

## The scope of artificial intelligence biology analysis for novel anticancer target investigations

**Fig. 2:**
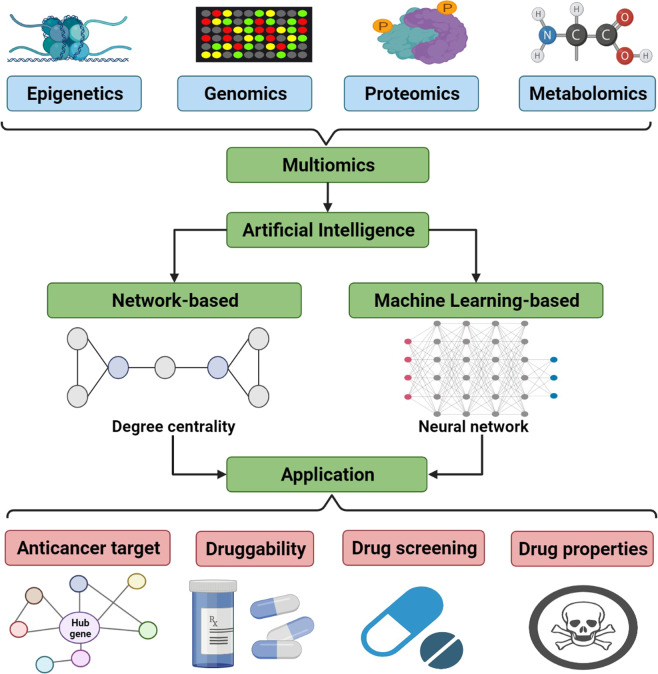
Artificial intelligence to integrate multiomics data (e.g., epigenetics, genomics, proteomics, and metabolomics) for cancer therapeutic targets identification. (Created with BioRender.com)

Recently, the rapid development of cancer-related multiomics technologies^34–36^ has been one of the most important factors for artificial intelligence biology analysis to explore novel anticancer targets^37–39^. Figure 2 classifies these technologies into five aspects: epigenetics, genomics, proteomics, metabolomics, and multiomics integration analysis. Furthermore, Table 1 lists the related major diseases, drug targets, genomics, and network databases commonly used in multiomics integration analysis for these five aspects. Next, we will detail these five aspects.

**Table 1:** Commonly used repositories related to human diseases, drug targets, genomics, and biological networks

Database name	Description	Web link	Ref
Disease			
Online Mendelian Inheritance in Man (OMIM)	A comprehensive, authoritative, and timely knowledgebase of human genes and genetic disorders	http://www.omim.org/	^40^
Pathologisch Anatomisch Landelijk Geautomatiseerd Archief (PALGA)	A database of histopathology and cytopathology was stored.	https://www.palga.nl	^41^
Drug Target			
DrugBank	DrugBank is a web-enabled database containing comprehensive molecular information about drugs, their mechanisms, their interactions, and their targets.	https://www.drugbank.ca/	^42^
Therapeutic Targets Database (TTD)	A database to provide information about the known and explored therapeutic protein and nucleic acid targets, the targeted disease, etc.	http://db.idrblab.net/ttd/	^43^
PubChem	PubChem is an open repository for chemical structures and their biological test results.	http://pubchem.ncbi.nlm.nih.gov	^44^
ChEMBL	ChEMBL is an open data database containing binding, functional and ADMET information for many drug-like bioactive compounds.	https://www.ebi.ac.uk/chembldb	^45^
Genomics Data			
Gene Expression Omnibus (GEO)	GEO is a public functional genomics data repository. Array- and sequence-based data are accepted.	https://www.ncbi.nlm.nih.gov/geo/	^46^
The Cancer Genome Atlas (TCGA)	TCGA contains clinical data of various human cancers, genomic mutations, mRNA expression, miRNA expression, methylation, etc.	https://www.cancer.gov/about-nci/organization/ccg/research/structural-genomics/tcga	^47^
Cancer Cell Line Encyclopedia (CCLE)	A compilation of gene expression, chromosomal copy number and massively parallel sequencing data from 947 human cancer cell lines.	https://sites.broadinstitute.org/ccle	^48^
ENCyclopedia Of DNA Elements (ENCODE)	ENCODE has systematically mapped regions of transcription, transcription factor association, chromatin structure, and histone modification.	https://www.encodeproject.org/	^49^
Catalogue Of Somatic Mutations In Cancer (COSMIC)	COSMIC curates comprehensive information on somatic mutations in human cancer.	http://www.sanger.ac.uk/cosmic	^50^
Biological Network			
Search Tool for the Retrieval of Interacting Genes/Proteins (STRING)	A database of known and predicted protein interactions	http://string-db.org/	^51^
Gene Ontology (GO)	The world’s largest source of information on the functions of genes.	http://www.geneontology.org/	^52^
Kyoto Encyclopedia of Genes and Genomes (KEGG)	A collection of databases dealing with genomes, biological pathways, diseases, drugs, and chemical substances	http://www.genome.jp/kegg/	^53^

Epigenetics analyses the reversal modifications of DNA or DNA-related proteins^54^. These modifications affect gene expression without changing the DNA sequence^54^. Investigating epigenetic data through artificial intelligence is not only important for elucidating fundamental mechanisms of cancer but also necessary for the design of targeted therapeutics. For example, Wilson et al.^55^ took advantage of information-rich transcriptomic and epigenetic data to study regulatory networks surrounding histone lysine demethylation and highlighted the importance of epigenetic regulators in mitogenic control and their potential as therapeutic targets, which showed that epigenetic regulators such as KDM1A, KDM3A, EZH2, and DOT1L^56^ are critical in oncogenesis and drug resistance.

Genomics aims to characterize the function of every genomic element of an organism by using genome-scale assays such as genome sequencing^57^. Applications of genomics include finding associations between genotype and phenotype^58^, discovering biomarkers for patient stratification^59^, predicting the function of genes^60^ and charting biochemically active genomic regions such as transcriptional enhancers^49^. Recent developments in network-based biology analysis methods, such as sequence-similarity networks, genome networks, and gene family networks, have significantly improved the usability of molecular datasets in comparative genomics analysis^61^. These network methods collect expression and interaction data in the beginning and then transform them into interpretable biological processes^62,63^, leading to the identification of tumour subtypes and the discovery of drug targets^64^.

For example, Medi et al.^65^ integrated gene expression profiles into genome-scale molecular networks to identify novel therapeutic targets for cervical cancer, including receptors, microRNAs (miRNAs), transcription factors (TFs), proteins (e.g., CRYAB, CDK1, PARP1, WNK1, GSK3B, and KAT2B), and metabolites (arachidonic acids). Laura et al.^66^ developed a network-based biology analysis workflow that integrates different layers of genomic information, including transcription factor cotargeting, miRNA cotargeting, protein–protein interaction and gene coexpression, into a biological network. Then, the authors applied a consensus clustering algorithm (An ML-based biology analysis algorithm that divide the network into sub-modules with different functions)^67–73^ on identified network communities to discover cancer driver genes, which demonstrated that F11R, HDGF, PRCC, ATF3, BTG2, and CD46 could be oncogenes and promising markers for pancreatic cancer.

For proteomics, proteomic experiments are performed for annotation and correlation of genome sequences, quantitation of protein abundance, detection of posttranslational modifications, and identification of protein-protein interactions (PPIs)^74^. PPIs not only play fundamental roles in structuring and mediating biological processes but also have been widely used for proteomics data analysis^75^. For example, Vinayagam et al.^37^ analysed the human PPI interaction network to identify indispensable proteins that affect the controllability of the network with control theory^76^, which shows that if a system can be driven from any initial state to any desired final state in finite time with a suitable choice of inputs, the system is controllable. By changing the number of driver nodes in the network upon removal of that protein, the hub can be classified as “indispensable” “neutral” or “dispensable”, which correlates with increasing, no effect, or decreasing the number of driver nodes in the network upon removal of the key protein. The evidence shows that these indispensable proteins are primary targets of disease-causing mutations, viruses, and drugs.

Furthermore, analysing data from 1,547 cancer patients revealed 56 indispensable genes in nine cancers. 46 of these genes were associated with cancer for the first time, demonstrating the ability of intelligent network controllability analysis to identify novel disease genes and potential drug targets^77^. Moreover, Valle et al.^78^ developed a network-based biology analysis framework to compute the proximity between polyphenol targets and disease proteins. The calculated results indicated that the diseases whose proteins are proximal to polyphenol targets have significant gene expression changes, while the diseases whose proteins are distal to polyphenol targets have no such change. The network relationship between disease proteins and polyphenol targets provides not only a computing method to reveal the effect of polyphenols on diseases but also a basis to identify novel anticancer targets.

Metabolomics is routinely applied for biomarker discovery by profiling metabolites in biofluids, cells and tissues^34^. Because of the inherent sensitivity of biotechnology, subtle alterations in metabolic pathways can be detected to provide insights into the mechanisms that underlie various physiological conditions and cancer processing^34^. Owing to innovative developments in network biology, researchers employ biological networks to perform metabolomic analyses and provide us with a systems-level understanding of the role that metabolites play in cancer.

For example, Basler et al.^79^ proposed an effective network-based biology analysis framework for the systematic study of flow control and identification of driver reactions in large-scale metabolic networks. They found that the driver reactions were under complex cellular regulation in Escherichia coli, suggesting their preeminent role in facilitating cellular control. Correlation statistics indicate that the driven response plays an important role in inhibiting tumour growth and represents a potential therapeutic target.

For multiomics integration analysis, addressing the complexity of tumour-host interactions requires an approach to handle integrative omics data^80^. Compared to single omics studies, multiomics data provide researchers with various and interconnected molecular profiles to study carcinogenesis^80^. Thus, integrated multiomics datasets in a network structure to artificial intelligence biology analysis has emerged as a powerful tool to fully appreciate the complex interlayer regulatory interactions in cancer progression. Such an approach allows us to benefit from prior information that can be summarized and presented in networks, thereby providing us with insights into carcinogenesis from an overall perspective^81^.

For example, Gov et al.^82^ first performed comparative analyses of transcriptome data, and then identified common and tissue-specific reporter biomolecules such as genes, receptors, membrane proteins, TFs, and miRNAs. Second, they used the interactions among receptors, TFs, miRNAs, and their targeted DEGs to reconstruct a tissue-specific network for ovarian cancer and used network-based biology methods to identify interaction hubs. Finally, GATA2 and miR-124-3p were identified as hub nodes, suggesting that they are potential biomarkers for ovarian cancer.

## The principles and theories for commonly used artificial intelligence biology analysis algorithms

This study divides these commonly used artificial intelligence biology analysis algorithms into two categories. One is network-based biology analysis algorithm, including shortest path^83^, module detection^84^, and network centrality^85^; the other is ML-based biology analysis algorithm including decision tree^86–88^ and deep learning models^89–91^.

### The principles and theory of network-based biology analysis algorithms

Biological networks are efficient in integrating complicated biological data, because they can capture the property of biological entities and their relationships^92^. Mathematically, a network can be represented as a graph *G* = (*V*, *E*) where V and E are a set of nodes (vertices) and edges, respectively. Nodes in biological networks can represent proteins, genes, diseases, and drugs and edges in the network represent various biochemical physical or functional interactions between nodes. Therefore, network-based biology analysis algorithms focuses on identifying therapeutic targets and discovery of novel drugs for cancer from molecular networks such as protein-protein interaction networks^75^, gene regulatory networks^93^, metabolic networks^94^, and drug-drug interaction networks^95^.

Computational biologists have developed several network-based biology analysis algorithms to effectively process and analyze non-ordered or non-Euclidean data in biological networks, which can perform tasks such as link prediction^96^, node ranking^85^, network propagation^97^, network modularization^98^, and network control^99^. Here, we briefly review and discuss the shortest path algorithm, module detection algorithm, and node prioritization methods using node centrality in identifying cancer therapeutic targets and discovering drugs.

#### Tthe shortest path algorithm

**Fig. 3:**
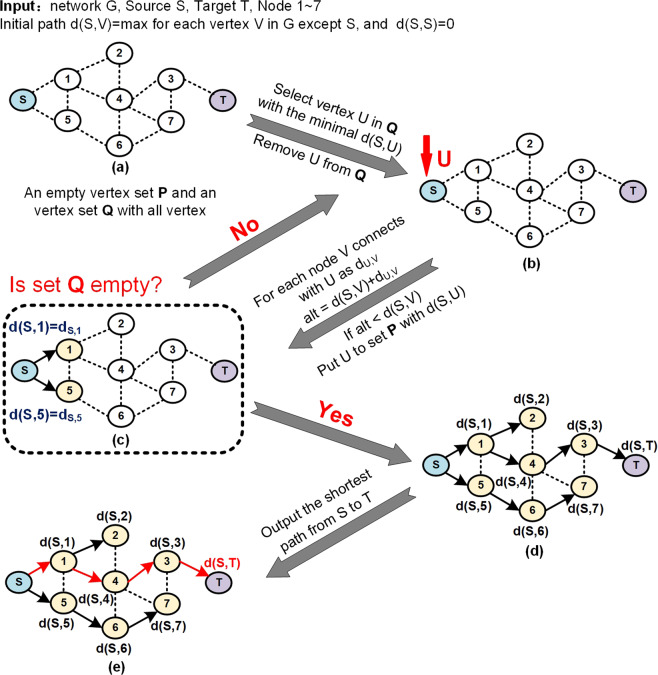
The flow chart of the shortest path algorithm. The red paths in the bottom network are the identified shortest path from node *S* to *T*

The shortest path algorithm, one of network link algorithm, is used to intelligently identify the shortest connection between two genes or proteins in a graphical model that represents a cellular network^100,101^. The algorithm is illustrated in Fig. 3 and Algorithm 1. The shortest distance for a given network is calculated by Eq. (1):

1 $$d(S,T) = \mathop {{\min }}\limits_{K \in V} \;d(S,K) + d_{K,T}$$

Here, *S* and *T* stand for the source and target node, respectively*. d(S,T)* is the length of the shortest path from node *S* to *T*. *V* is a set of network nodes. *K* stands for a node in the network, and *d*_*K,T*_ represents the lengths of possible paths connecting nodes *K* and *T*.

##### Algorithm 1

The shortest path algorithm^102^

**Table:** 

1:	**Input**: Network G, Source S, Target T, Nodes
2:	create an empty set P and a set Q contains all nodes
3:	**for each** vertex V **in** Network:
4:	d(S,V) ← infinity
5:	d(S,S) ← 0
6:	**do**:
7:	U ← vertex in Q with minimal d(S,U)
8:	remove U from Q
9:	**for each** vertex V in Q that is connected with U:
10:	alt ← d(S,U) + d_U,V_
11:	**if** alt < d(S,V):
12:	d(S,V) ← alt
13:	add U to the set P
14:	**until** Q is empty
15:	**Output**: the shortest path from S to T

The shortest path algorithm has been widely used to determine regulatory paths in cancer networks^103,104^ and then discover the key targets on the paths^105^. For example, Li et al.^106^ first identified a set of six genes that can distinguish colorectal tumours from normal adjacent tissues using the maximum relevance minimum redundancy approach^107^. The method ranks genes according to their relevance to the class of samples concerned while considering the redundancy of genes. Those genes that had the best trade-off between the maximum relevance to the sample class and the minimum redundancy were considered “good” biomarkers. Then, the authors applied the shortest path algorithm among the six genes in a PPI network underlying cancer and identified 15 shortest paths between any two genes of the gene set. Last, they found 35 genes on the identified shortest paths and ranked them according to their betweenness^108^. The results showed that androgen receptor (AR), a ligand-dependent transcription factor, is ranked as the top gene, suggesting its involvement in colon carcinogenesis through regulating the proliferation and differentiation of tumour cells^109^.

Additionally, Chen et al.^105^ used a network-based biology analysis method, SAM (Significance Analysis of Microarrays)^110^, to analyse omics data and identified 153 differentially methylated CpG sites and differentially expressed molecules, including 42 miRNAs and 1,373 protein-coding genes. The authors first used the differentially expressed genes from the STRING database^111^ to construct a PPI network. Then, they searched all the shortest paths connecting dysfunctional genes to identify potential cancer driver genes. Next, they ranked the genes by a permutation test and their network properties, such as betweenness and interaction scores. The top-ranking genes at different levels (i.e., methylation level, miRNA level, mutation level, and mRNA level) were regarded as driver genes of lung adenocarcinoma. Among these cancer driver genes, some appeared to be top candidates at different levels, suggesting their multifaceted contribution to lung carcinogenesis.

Above all, the shortest path algorithms^100,101^ can help us efficiently identify regulatory paths in networks, allowing us to identify potential genes that are proximate to known cancer genes and thereby important for tumorigenesis. However, due to the complexity of the disease, potential cancer genes are not always on the identified shortest paths^106^, revealing the limitations of such algorithms. To resolve this issue, Lu et al.^112^ proposed a random walk with restart algorithm method and identified 298 potential CRC-associated genes, which is more effective and accurate than the shortest path algorithm proposed by Li et al.^106^. In particular, the computing efficacy of the shortest path algorithm could be compromised by large networks and their search strategies^112^.

#### The module detection algorithm

**Fig. 4:**
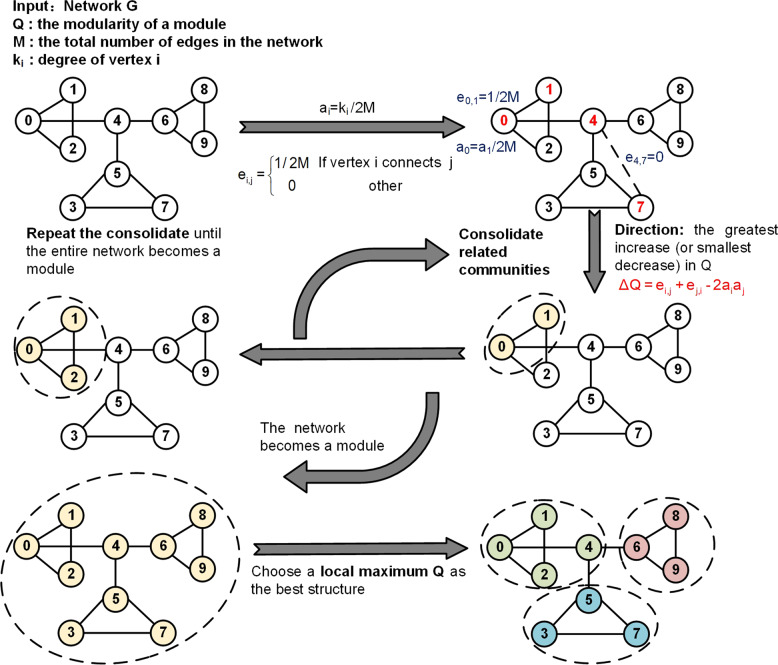
The flow chart of the module detection algorithm

Cancers usually result from disruption of interactions of key regulatory genes with their partners^81,113^. Module detection algorithms^114^, one of network propagation algorithm, identify communities of cancer genes in complex networks^115^ by analysing their topological structures (Fig. 4 and Algorithm 2). Here, we explain and illustrate the commonly used modularity maximization algorithm^116^, which identifies network modules with the maximum modularity coefficients by Eq. 2.

2 $$Q = \frac{1}{{2M}}\mathop {\sum}\limits_{i,j\, \in \,V} {[A_{ij} - P_{ij}] \cdot \delta _{C_i,C_j}}$$

where *Q* represents the modularity coefficient of an identified module, *M* is the total number of edges in the network, *A*_*ij*_ is the adjacency matrix, and *P*_*ij*_ represents the expected number of edges between nodes *i* and *j*. *C*_*i*_ or *C*_*j*_ represents the module to which node *i* or node *j* belongs. If *i* and *j* belong to the same module, $\delta _{C_i,C_j} = {{{\mathrm{1}}}}$; otherwise, $\delta _{C_i,C_j} = {{{\mathrm{0}}}}$. The identified modules are a group of genes that are supposed to have a similar biological function, such as promoting or inhibiting tumourigenesis.

##### Algorithm 2

Module detection algorithm.

**Table:** 

1:	**Input**: Network G
2:	M ← the total number of edges in the Network
3:	**for each** vertex i **in** Network:
4:	i ← a single module
5:	k_i_ ← degree of vertex i
6:	a_i_ ← k_i_/2 M
7:	**for each** edge **in** Network:
8:	**if** vertex i connects j:
9:	e_i.j_ ← 1/2 M
10:	**else**:
11:	e_i.j_ ← 0
12:	**do**:
13:	ΔQ ← e_i.j_ + e_j,i_-2a_i_a_j_
14:	consolidate related communities
15:	direction ← the greatest increase (or smallest decrease) in Q
16:	**until** the entire network becomes a module
17:	**Output**: the module with a local maximum Q

Currently, many researchers employ module detection algorithms to intelligently identify potential therapeutic targets for cancer^117–119^. For example, Ghiassian et al.^120^ used the DIseAse MOdule Detection (DIAMOnD) method^121^ to identify the local modules within the interconnected map of molecular components. They found that disease-related genes were significantly enriched in highly overlapping modules, which indicated that the predicted modules may help identify new anticancer targets. Of note, since the results of module detection algorithms depend mainly on network structures, the identified modules may vary for the same disease network with slightly different topology^85,117^.

Since potential drug targets may exist in different network modules, we can make use of the correlation between modules to identify reliable cancer treatment targets^81^. Therefore, Wang et al.^122^ proposed the seed connector algorithm (adding a few extra hidden nodes as much as possible to link disease proteins) by considering the interactions among cancer-associated proteins. First, this algorithm starts with known seed proteins and induces a loosely connected subnetwork consisting of only seed proteins. Second, Wang et al. sequentially select such proteins as seed connectors that maximally increase the size of the largest connected component of the subnetwork until there is no additional protein that can be selected as a seed connector. Finally, the cancer modules are pinpointed.

While these aforementioned algorithms^122–124^ can intelligently identify meaningful functional modules from network topologies, it may be difficult to capture disease modules^125^. One possible reason is that disease proteins do not constitute particularly densely connected subgraphs but agglomerate in specific large regions of the network. For this reason, Tripathi et al.^126^ considered analysing the patterns of connectivity in a disease module to be an effective way to understand the properties of disease modules.

#### The node centrality

Node centrality measures the importance of nodes and is suitable to intelligently locate key nodes with important biological functions for network biology^127^.

Usually, we listed four types of node centrality as follows: (1) As the simplest form of network centrality, degree centrality is the number of nodes directly connected to the network^127,128^; (2) Coreness centrality considers both the degree of nodes and their positions in a network^129^; (3) Betweenness centrality of a node is the probability for the shortest path between two randomly chosen nodes to go through that node, and it determines the actor that controls information among other nodes by connecting paths^130^; (4) Eigenvector centrality^131^ not only considers the number of edges and the position of nodes but also the impact of adjacent nodes on the interactive network.

**Fig. 5:**
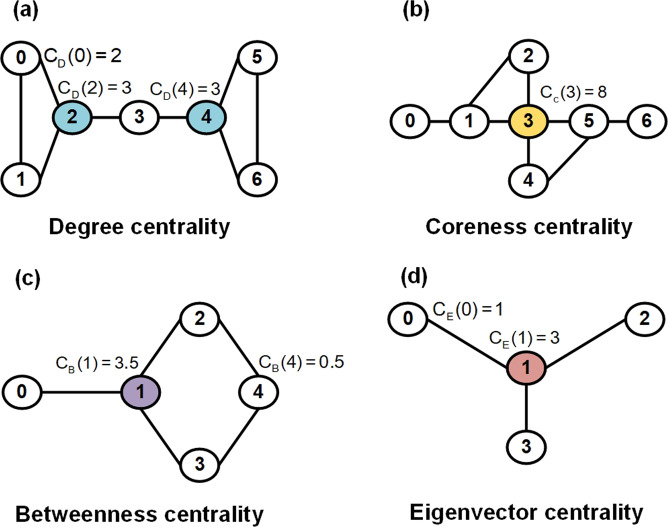
Four types of node centralities of biological networks. (a) Degree centrality; (b) Coreness centrality; (c) Betweenness centrality; (d) Eigenvector centrality

Table 2 shows the formulas for node centrality computing. Figure 5(a–d) illustrates the above four types of node centrality, and Algorithm 3 presents the pseudocode to compute four types of node centrality.

**Table 2:** The formula to compute degree centrality, coreness centrality, betweenness centrality and eigenvector centrality

Node centrality	Formula	Description	Eq.
Degree centrality	$C_D(i) = d_i$	d_i_ is the degree of vertex i.	(3)
Coreness centrality	$C_C(i) = \mathop {\sum}\nolimits_{j \in N(i)} {ks(j)}$	Vertex j belongs to the neighbours of vertex i, ks(j) is the k-shell index of vertex j.	(4)
Betweenness centrality	$C_B(i) = \mathop {\sum}\nolimits_{j < k} {g_{j,k}(i)/g_{j,k}}$	g_j,k_ is the number of all shortest paths between j and k, g_j,k_(i) is the number of shortest paths between j and k containing i.	(5)
Eigenvector centrality	$C_E(i) = \frac{{{{\mathrm{1}}}}}{\lambda }\mathop {\sum}\nolimits_{{{{\mathrm{j}}}} \in G} {{{{\mathrm{a}}}}_{i,j}x_j}$	if vertex i is linked to vertex j, a_i,j_ = 1, x_j_ is the degree of vertex j, λ is a constant.	(6)

##### Algorithm 3

The algorithm of degree centrality, coreness centrality, betweenness centrality and eigenvector centrality.

**Table:** 

1:	**function1** Degree centrality:
2:	**Input**: Network G
3:	**for each** vertex i **in** Network:
4:	d_i_ ← the number of ties that vertex i has
5:	C_D_(i)=d_i_
6:	**Output**: C_D_(i)
7:	**function2** Coreness centrality:
8:	**Input**: Network G
9:	**for each** vertex i **in** Network:
10:	N(i) ← the set of the neighbours adjacent to vertex i
11:	**for each** vertex **j** in N(i):
12:	ks(j) ← the k-shell index of vertex j
13:	C_C_(i) ← C_C_(i) + ks(j)
14:	**Output**: C_C_(i)
15:	**function3** Betweenness centrality:
16:	**Input**: Network G
17:	**for each** vertex i **in** Network:
18:	**for each** vertex j **in** Network:
19:	**for each** vertex k **in** Network:
20:	**if** j < k:
21:	g_j,k_ ← number of all shortest paths between j and k
22:	g_j,k_(i) ← number of shortest paths between j and k containing i
23:	C_B_(i) ← C_B_(i) + g_j,k_(i)/g_j,k_
24:	**Output**: C_B_(i)
25:	**function4** Eigenvector centrality:
26:	**Input**: Network G
27:	**for each** vertex i **in** Network:
28:	**for each** vertex j **in** Network:
29:	**if** vertex i is linked to vertex j:
30:	a_i,j_=1
31:	**else**:
32:	a_i,j_=0
33:	x_j_ ← the degree of vertex j
34:	C_E_(i) ← C_E_(i)+ 1/λ ∙ a_i,j_x_j_
35:	**Output**: C_E_(i)

As described in Fig. 5(a) and Eq. 3, the degree centrality of node 2 is 3 (C_D_ (2) = 3) because node 2 interacts with nodes 0, 1, and 3. We demonstrated that highly connected nodes or hubs are more likely to be essential^127^. Because the more direct connections a node has, the greater the impact that the node can exert on the network^132^, we can utilize the degree centrality of nodes to identify cancer therapeutic targets.

For example, Zhang et al.^133^ predicted that hypoxia inducible factor-1α (HIF-1α) and prolyl 4-hydroxylase beta polypeptide (P4HB) may be considered potential biomarkers of gastric cancer by constructing a PPI network. Nevertheless, not only Jalili et al.^130^ suggested that high connectivity does not necessarily imply its essentiality, but also Kitsak et al.^129^ argued that the location of nodes is more significant than the immediate neighbours to evaluate its spreading influence because degree centrality considers only direct interactions of a node but not its impact on other nodes, resulting in low accuracy for target prediction compared to other methods such as coreness centrality^134^.

As shown in Fig. 5(b) and Eq. 4, the coreness centrality of node 3 is 8 (C_C_ (3) = 8) because the neighbours adjacent to the labelled vertex (3) are vertex (1), vertex (2), vertex (4) and vertex (5), and these four nodes belong to a 2-shell. Coreness centrality is an advanced form of node centrality because it considers both the degree of nodes and their positions in a network to quantify the importance of nodes in a network^129^. A node with a greater coreness means that the node is located in a more central place and is much more influential in network propagation than the nodes with high-degree but less coreness^129^. Among them, the most classic method to calculate the coreness centrality of network nodes is the k-core decomposition method^135^, which decomposes the network iteratively according to the remaining degree of the nodes.

For instance, Li et al.^136^ employed the k-core decomposition method to obtain the coreness of the PPI network. Subsequently, the targets were screened for topological importance. Then, the major hubs in the hub interaction network were determined, and a total of 62 major hubs were identified, including 11 indirubin (EGFR, JAK2, ERBB2, CHUK, CDK5, KIF11, DRD2, CDK3, HTR1A, JAK3 and TYK2) and derivative targets and 51 differentially expressed genes (DEGs) for imatinib resistance. These 11 major hubs were closely related to DEGs that were resistant to imatinib. Indirubin and its derivatives may inhibit imatinib resistance through the regulation of these genes to treat chronic myeloid leukaemia (CML).

Described by Fig. 5(c) and Eq. 5, the betweenness centrality of node 1 is 3.5 (C_B_ (1) = 3.5) because there are four node pairs contributing to node one (g_0,2_(1)/g_0,2_(1) = 1, g_0,3_(1)/g_0,3_ = 1, g_0,4_(1) / g_0,4_ = 1, and g_2,3_(1)/g_2,3_ =0.5). Betweenness centrality is based upon the frequency with which a node lies between the shortest path of all other possible pairs of nodes within a network and identifies the gatekeepers that control communication of nodes in the network^130^.

For example, Taylor et al.^137^ used betweenness centrality analysis to identify intermodular hub proteins and intramodular hub proteins in the breast cancer network. The identified proteins may serve as an indicator of breast cancer prognosis. Moreover, Raman et al.^138^ computed degree, betweenness, and closeness indices in PPI networks for 20 organisms and showed that the degree and betweenness centralities of nodes correlate with their lethality in many organisms.

As described in Fig. 5(d) and Eq. 6, the eigenvector centrality of node 1 is 3 (C_E_ (1) = 3) because node 1 is connected to nodes 0, 2 and 3 (a_1,0_, a_1,2_ and a_1,3_ equal 1, respectively), and the degree of x_0_, x_2_ and x_3_ equals 1, respectively. Eigenvector centrality considers not only the number of edges and the position of nodes but also the impact of adjacent nodes on a network.

For example, Mallik et al.^139^ first identified differentially expressed and methylated genes in uterine leiomyoma tumours and then found TFs and miRNAs that regulate the expression of these genes. Subsequently, they reconstructed a network that comprised the genes, TFs, and miRNAs and then used eigenvector centrality to identify potential biomarkers. They specified that PTGS2 and TACSTD2 are potential novel biomarkers, since both genes are downregulated and hypermethylated in the tumour.

Moreover, several researchers have attempted to integrate more than one centrality index to increase the efficiency of the node centrality algorithm. For instance, Chen et al.^140^ used the differentially expressed proteins of prostate cancer (PC) to construct a PPI network. Then, they integrated the connectivity degree, betweenness centrality, and closeness centrality of nodes to evaluate critical nodes to identify the core module of the PPI network. Finally, they identified SLC2A4 and TUBB2C as important proteins regulating the pathogenesis of cancer, suggesting the proteins involved in biological processes and pathways as potential targets for PC diagnosis and treatment. In addition, Aamri et al.^141^ constructed a gene-gene-interaction network for the entire human genome and then applied betweenness, closeness, eigenvector, and degree centrality metrics to rank the central genes of the network to identify possible cancer-related genes. The results showed that the average precision for identifying breast, prostate, and lung cancer genes varied between 80–100%.

Although highly connected nodes in the network architecture are essential, recent studies point out that integrating the prior knowledge of cancer into centrality indices can accurately identify anticancer targets^130^. For this reason, Jiang et al.^142^ developed a network-based biology analysis method, named NEST, which predicts essential proteins according to the expression levels of their interacting partners in a network. Additionally, the results showed that NEST significantly outperformed the classic centralities on gene essentiality prediction and functional screen result enhancement.

### Machine learning-based biology analysis algorithms

Machine learning (ML) algorithm is a subset of AI algorithms that can learn from data, therefore removing the need for explicit instructions on how to do certain tasks^15^. The key to identify therapeutic targets and discover drugs using ML-based biology analysis is to make use of network features in biological networks. The network features include the topological features (such as node centrality, interaction, local structure, subgraph, network propagation results, and network-based structure similarities) and the biological information that is embedded in network nodes (such as the gene expression profile, gene mutation frequency, and gene functional annotation).

Here, we introduce two classical ML-based algorithms: one is the decision tree algorithm, which selects significant topological features for cancer; the other is deep learning, which uses the network features to identify cancer targets and discover drugs.

#### The decision tree algorithm

**Fig. 6:**
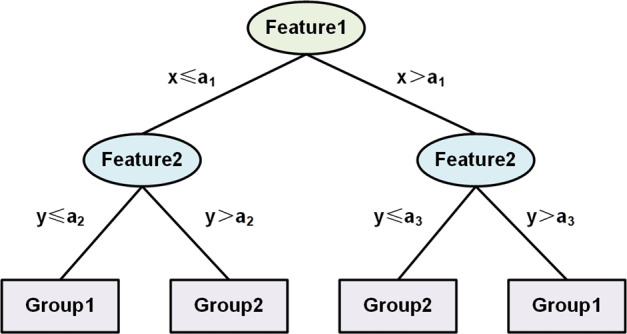
An illustration of a simple decision tree model

A decision tree is a supervised classification algorithm^143^ with three steps: feature selection, decision tree generation, and decision tree pruning^86–88^. Figure 6 shows how to classify a set of samples into two groups using the decision tree algorithm.

In the network-based biology analysis, network topology features^88^ are usually integrated into a decision tree to classify gene-phenotype associations for cancers^144–146^ to select significant topological features for cancer.

For instance, Ramadan et al.^147^ extracted thirteen network topological features (Table 3) from a publicly available gene co-expression network and a PPI network of breast cancer. Then, to assess the significance of topological measurements associated with breast cancer, they used Decision Tree Bagger^156^ to classify breast cancer gene-phenotype associations. The importance of each topological measure was then evaluated using a score that combines the accuracy of breast cancer classification and the Gini index^148^ (Table 3). The computed scores of the top five identified features (i.e., structural holes, node degree, node coreness, *k*-Step Markov and subgraph) outperformed the others, and they were selected as key features for the classification of breast cancer phenotype-gene associations.

**Table 3:** Thirteen network topological features for decision tree classification^147^. The score is a combination of the classification accuracy and the Gini index^148^

Topological measures	Concept	Score
Structural holes^149^	Rank nodes by their connectivity and lack of redundancy	13.37
Node degree	The number of connections of a node	13.36
Node coreness	Considers both the degree of nodes and their positions in a network	12.05
k-Step Markov^150^	The probability that a random walk of length k makes the system reach a certain vertex	10.47
Subgraph^151^	The number of times a given vertex participates in different connected subgraphs of a network	10.36
Within–module z-score^152^	Measure how nodes are related.	8.88
Katz status index^153^	Rank a vertex as highly important if many nodes are connected to it.	8.64
Closeness	The average length of the shortest path between nodes	8.18
Proximity prestige	The average shortest path length of a node	8.12
Eigenvector centrality	The influence of directly adjacent nodes on central node	8.09
Betweenness	A node acts as a bridge along the shortest path between two other nodes	7.93
Bary centre score^154^	Rank the nodes by the total shortest path of the vertex	5.70
Clustering coefficient^155^	Measure the degree of cohesiveness	0.15

Although the decision tree algorithm can help us select key network features, it usually has the overfitting problem when too many features exist in the network^157^, which significantly decreases the classification and prediction on independent testing^157^.

At present, there are two commonly used methods to resolve overfitting caused by the decision tree algorithm. One method is using dimension reduction^157^ and pruning strategy^86^ to improve the classification accuracy by feature reduction; the other is employing the random forest algorithm^158^, an ensemble algorithm with multiple decision trees. The random forest algorithm adopts a bagging strategy, which has higher accuracy and reliability than the classical decision tree algorithm^159^.

For example, Toth et al.^160^ used the random forest algorithm to predict the aggressive behaviour of prostate cancer. Their methylation-based classifier demonstrated excellent performance in discriminating prognosis subgroups of the test set (Kaplan-Meier survival analyses with log-rank *p* value < 0.0001) with an AUC value of 0.95^161^ for the sensitivity analysis. Finally, the experimental verification showed that the loss of ZIC2 protein expression was associated with poor prognosis and correlated with a significantly shorter time to biochemical recurrence.

**Fig. 7:**
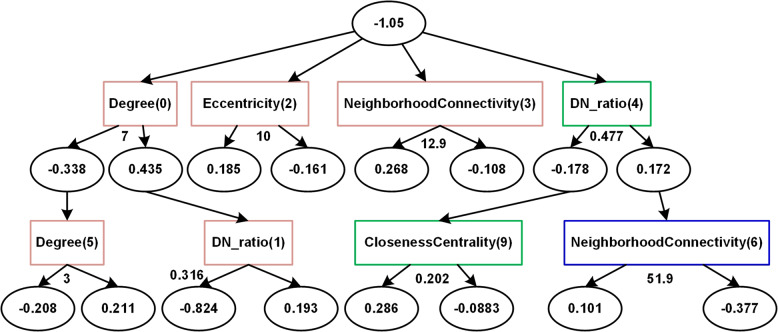
An example of an ADTree model. The root nodes indicate the ratio between positive and negative class examples. The numbers in parentheses within each decision node (rectangles) indicate the order in which the rule was found. The amount of node conservation between each of the trees is indicated by the colour of the box. Ovals (prediction nodes) contain the value for the weighted vote. The numbers next to the arrows correspond to the threshold for the prediction

In addition to the overfitting problem, it is difficult for decision trees to visualize the complicated classification procedure^146^. Recently, the alternating decision tree (ADTree)^162^ has made the classification procedure intuitive and easy to understand by adding an intuitive graphical model, and the algorithm builds decision trees over a user-defined number of iterations using confidence-rated boosting, so it returns both a class label and a score that measures confidence in the classification, as shown in Fig. 7 and Algorithm 4.

For example, Carson et al.^146^ used ADTree to classify proteins in a breast cancer network. As indicated in Fig. 7, the most effective attributes to distinguish disease and non-disease proteins are node degree, disease neighbour ratio, eccentricity, and neighbourhood connectivity, which was proven by Hao et al.^163^ and Zhang et al.^164^.

##### Algorithm 4

The algorithm of ADTree model^165^

**Table:** 

1:	**Input**: labelled dataset
2:	root node ← the bias in the dataset
3:	**for each** decision node **in** the tree:
4:	a_i_ ← attribute value
5:	t_i_ ← threshold
6:	**for each** decision node **in** the tree
7:	**if** (the decision node has a parent node):
8:	**if** a_i_ ≥ t_i_:
9:	**return** the score of the prediction node for the left path
10:	**else**:
11:	**return** the score of the prediction node for the right path
12:	**else**:
13:	**return** 0
14:	s ← the sum of all scores acquired
15:	**if** s > 0:
16:	**Output**: the positive class
17:	**else**:
18:	**Output**: the negative class

Although the decision tree, random forest and ADTree^86–88,158^ demonstrate the tendency to identify such proteins that are well annotated and studied for cancer, these methods are subject to producing local optimal solutions. Therefore, Chen et al.^143^ proposed using the decision tree classifier based on particle swarm optimization^166^ to avoid falling into the trap of local minima by adding randomness to optimize the number of features and detection accuracy of cancer treatment targets. Furthermore, the gradient boosting decision tree^167^ is a very flexible and scalable method to classify network nodes for future study.

#### The deep learning algorithms

Deep learning is a subfield of machine learning, and the origin of neural networks sets the stage for the emergence of deep learning models^168^. Deep learning model is a neural network composed of complex structures and nonlinear transformations^90,91^ that attempts to model high-level abstractions of data using multilayer neurons. Through training and iteratively updating its hyperparameters (Eq. 7), the initial low-level feature representation (such as topological features and biological information) of samples is transformed into the high-level representation that shows the distinction between samples. The strength of deep learning is its ability to detect complex patterns in data, making it suitable to interrogate the biological networks that consist of complex, interdependent relationships among genes.

7 $$W_{{{\mathrm{k}}}} \to W_{{{{\mathrm{k}}}} + {{{\mathrm{1}}}}} = W_{{{\mathrm{k}}}} - \eta \frac{{\partial C}}{{\partial W_{{{\mathrm{k}}}}}}$$

*W*, *k*, and *C* are the weight, iteration, learning rate, and loss function, respectively.

Currently, there are many neural network models and complex functions for ML-based biology analysis. In this paper, we only present several commonly used neural networks (Table 4). Benefiting from the strong ability of neural networks in mining complex information on links or nodes, deep learning is a suitable method to identify potential cancer targets and discover drugs for cancer treatment in complex biological networks^175^. For example, Selvaraj et al.^176^ searched for therapeutic targets for lung adenocarcinoma in a network of protein-protein and protein-drug interactions and employed a neural network to identify candidate drugs, where phosphothreonine is predicted via molecular dynamics simulations to target the hub node MAPK1 in the network.

**Table 4:** Commonly used neural networks in ML-based biology analysis

Model	Characteristic	Application scenarios
Non-graph Neural Network		
DNN	Deep neural network (DNN), also called multi-layer perceptron, is a neural network with multi-layer hidden layer.	^169^
CNN	Convolutional neural network (CNN) obtains local information between input data by convolution.	^170^
Graph-based Neural Network		
GCN	Graph convolutional network (GCN) applied cconvolution in networks to obtain local information between nodes and neighbour nodes.	^171^
GAE	Graph autoencoder (GAE) uses autoencoder to extract the embedded features of the network.	^172^
GAN	Graph attention network (GAN) uses attention mechanism instead of convolution to obtain local or global information between nodes.	^173^
DeepWalk	DeepWalk is a network embedding model, which can represent the attributes of graph nodes as low dimensional and dense eigenvectors.	^174^

**Fig. 8:**
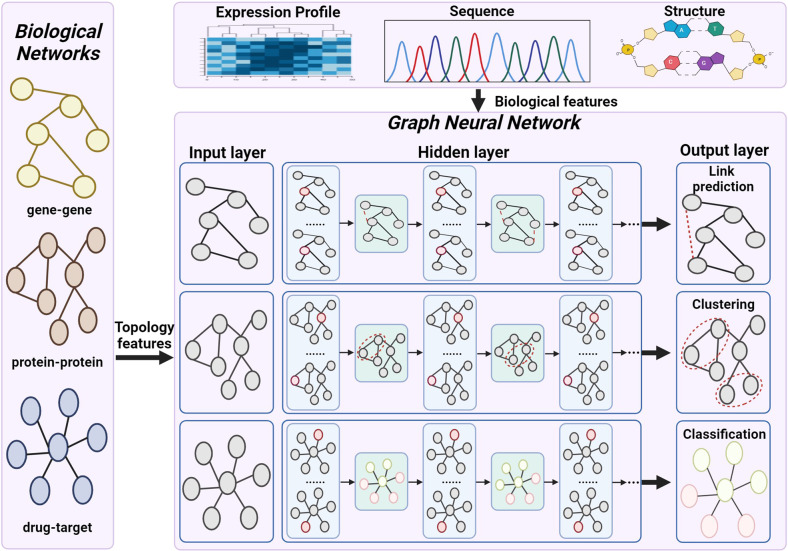
The illustration of graph-based neural networks for ML-based biology analysis. The graph-based neural networks take the topology of the biological networks data (such as gene-gene networks, protein-protein networks and drug-target networks) as input data. And then, the graph-based neural network realizes the functions of link prediction, classification and clustering by analyzing the biological information in the network topology. (Created with BioRender.com)

Currently, artificial intelligence biology analysis has benefited from the utilization of graph-based neural networks instead of commonly used non-graph neural networks such as CNN^170^ or DNN^169^, because graph-based neural networks can take the biological network structure as the input directly, learn an embedding that contains information about the neighbourhood of a target node in a graph, and analyse the biological network with neural networks technology. Figure 8 illustrates the basic flowchart of graph-based neural networks for the investigation of different properties of biological networks.

There are two advantages in using graph-based neural networks to identify cancer targets or discover drugs from biological networks.

1. Feature representation. Graph embedding^177^ is the core method to extract features in graph-based neural networks, which represent network nodes as a low-dimensional vector representation, preserving both network topology and node content information^178^. For example, Li et al^174^ proposed a similarity-based miRNA-disease prediction method that used DeepWalk, a graph embedding algorithm, to compute the topological similarities between two diseases nodes. The model extracts the disease node features in the disease-disease network based on the random walk algorithm, and significantly enhances the prediction performance by utilizing global network association information. For diseases nodes with similar features, if one of the diseases is associated with miRNA, the other is predicted to be associated with the miRNA.
  In addition, Zheng et al.^179^ proposed an attention-based graph neural networks (attention mechanism assigns different weight parameters to different targets through learning, so as to consider the importance of key targets locally and globally^180^) to learn the graph embedding feature (association scores) from piRNA-disease association network. The results showed that the predicted scores of piRNA-disease associations are positively correlated with the association probability between a piRNA and a disease, suggesting that piRNAs with closer distances to tumour genes in the network are more likely to be therapeutic targets of cancer.
2. Feature integration, which integrates the heterogeneous, noisy, nonlinear-related biological network information (such as node similarity, node interactions, upstream and downstream relationships) multi-views (such as drug molecular structures and drugs’ indications)^181^. For example, Ma et al.^172^ proposed a novel graph autoencoders model (GAE) to learn accurate and interpretable drug similarity measures from multiple types of drug properties. The GAE uses attention mechanism^180^ to integrate multi-view (multiple types of drug properties) from drug-drug interactions network and determines the weights for each view with respect to the similarity measure tasks for better explaining the contribution of drug properties to drug similarity. Due to the ability to integrate network data from multi-views and autoencoder structures, GAE can resist the noise interference in the data. Thus, graph-based neural networks are more robust and reliable in most application scenarios^182^.

Overall, deep learning can comprehensively explore features such as node degree, edge length, and module in biological networks^83–85,183^ to provide an accurate prediction for drug targets of cancer through artificial intelligence of multiomics data in complex biology networks^184^. However, there are still two key issues to be addressed. One is the interpretability of the models, which is critical for clinical adoption^185^. The other is how to demonstrate the generalizability of the approach^185^ and validate these approaches in the context of multi-institutional datasets. Therefore, these issues are actively being tackled from model interpretation, extraction of biological insights^186^ and model reproducibility^187^.

## The artificial intelligence biology analysis for biomedical applications

Because the wide and easy accessibility of high-throughput data in oncology has provided the basis for developing novel artificial intelligence methods and validating their capability to identify therapeutic targets, this section will focus on reviewing the biomedical applications from four perspectives. First, we present the artificial intelligence applications to identify novel anticancer targets. Second, we present the artificial intelligence applications to evaluate the druggability of potential target genes. Third, we show the artificial intelligence applications for drug discovery. Fourth, we show the artificial intelligence applications for drug property prediction.

### Identification of novel anticancer targets

Artificial intelligence biology analysis applications^188^ usually use omics data to build networks and identify co-expression modules of genes, proteins, metabolites, critical pathways between molecules, and key molecules in biological networks^189^. This study will introduce these applications from two perspectives: one is network-based biology analysis applications, and the other is ML-based biology analysis applications.

#### Network-based artificial intelligence for identifying novel anticancer targets

Network-based biology analysis applications firstly reconstruct networks by computing differential expressions of molecules and their correlations^190–193^. Then, gene set enrichment analysis are performed to identify network modules with different biological functions^194^. Finally, the identified network modules are used to discover key genes that are potential therapeutic targets (or biomarkers) for cancer. Here, we show the key target identification procedure by network-based biology analysis applications as follows.

**Fig. 9:**
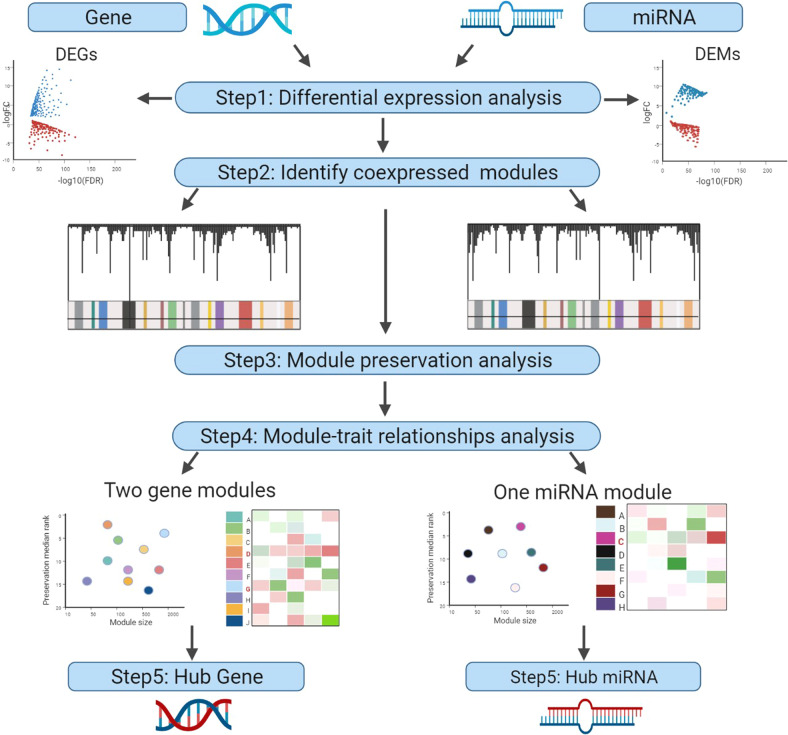
The workflow to identify novel anticancer targets by network-based. (Created with BioRender.com)

WGCNA^195^ is a commonly used network-based biology analysis application that uses various gene expression matrices as input. Then, WGCNA outputs different gene network modules and the core genes in the biological network. For example, Zhou et al.^196^ used WGCNA to analyse colorectal cancer data from TCGA (Fig. 9), which demonstrated that 11 hub genes and 5 hub miRNAs have predictive power for the prognosis of colorectal cancer patients by the following steps.

In Step 1, the correlation between all pairs of genes and miRNAs by differential gene expression analysis was calculated, and two similarity matrices were constructed. In Step 2, the adjacency matrix, which comes from similarity matrices, is transformed into a topological overlap matrix (TOM) by using TOM similarity, and then the coexpressed gene and miRNA modules are identified by using dynamic tree cutting^197^. In Step 3, after module preservation analysis, six gene modules were found to have strong stability, and one miRNA module was found to have low stability. In Step 4, they performed module-trait relationship analysis to further validate the module–clinical trait relationships, and two pathological stage-related gene modules and one pathological stage-related miRNA module were identified. In Step 5, hub genes and hub miRNAs were identified by calculating the module membership and gene significance.

Though network-based biology analysis methods are useful in identifying anticancer targets, they have some limitations, such as they cannot effectively handle multiomics data, leading to high false-positive rates of identified targets^42^. Developing comprehensive network-based biology analysis applications may resolve the problems and increase the precision for predicting cancer biomarkers^198^.

For example, Lai et al.^199^ deployed an integrated approach that combined network-based algorithms and RNA sequencing data to delineate miRNA-based strategies that enhanced DC (dendritic cell)-elicited immune responses. First, the authors performed RNA sequencing to obtain the protein-coding genes and miRNAs in relation to standard DCs. Then, they analysed miRNA-gene interactions at the pathway level and reconstructed regulatory networks underlying the immunological functions of DCs. Finally, they performed network-based prioritization of miRNAs by combining their expression profiles and strength of association with other protein-coding genes. Their analysis identified dozens of promising miRNA candidates, of which miR-15a and miR-16 are the most promising ones for increasing the immunogenic potency of DCs and therefore improving DC-based immunotherapy against cancer.

In summary, we consider that an increasing number of network-based biology analysis applications will be developed for novel anticancer targets identification in the distant future.

#### ML-based artificial intelligence for identifying novel anticancer targets

ML-based biology network analysis applications are applied to interrogate the large, complex data and thus identifying reliable potential novel targets as effective treatments of human diseases^200^. These ML-based biology analysis applications for novel anticancer targets identification consist of classification^201^, clustering^202^, neural networks^203,204^, and so on^205^. Here, due to the limit space of the review, we only focus on the ML-based biology network analysis applications for classifications and graph-based neural networks.

ML-based biology network analysis applications for classifications identify key targets by determining the key factors of classifications^206^. It considers specific biomarkers (such as gene or protein nodes) of the defined classes as key targets^206^. Recently, the classification-based applications and molecular profiling^207^, use genome-wide gene transcription profiles, protein expression profiles and/or mutational landscapes to make a more accurate classification of tumor subtypes and identify biomarkers for specific tumor types.

For example, Sinkala et al,^208^ applied classification analysis on networks to reveal subtypes of pancreatic cancer and their molecular characteristics. Firstly, the authors employed K-means clustering to the reverse phase protein array (RPPA), determined proteomics data with 45 high-purity pancreatic cancer samples, and then identified two clusters of samples.

Secondly, they compared their clustering results to other subtypes that have been reported in the literature for various other molecular data types (such as DNA methylation status, protein expression levels and expression levels of mRNAs and miRNAs), and then applied the similarity network fusion (SNF) to identify two-cluster and three-cluster solutions comprised 25 and 20 tumors. The SNF method solves the disparate clustering problem by constructing similarity networks of samples for each available molecular data type and then efficiently fuses these into one network that represents clustering based on all the underlying data.

Thirdly, they applied proteomics-based signaling pathway analysis to distinguish disease subtypes and found that, for tumors of the two major pancreatic cancer subtypes, oncogenesis may be primarily driven by perturbation in either SMAD4 or mTOR signaling pathways. Furthermore, they performed gene set enrichment analysis using the Gene Ontology database^52^ and found that pancreatic cancer subtypes classified by mRNA expression levels and DNA methylation statuses show differences in molecular functions in terms of mRNA.

Finally, given that different types of molecular data yield different patterns of tumor clustering, they attempted to identify a list of biomarkers that can differentiate the two tumor subtypes. Using neighborhood component analysis, they identified biomarker sets comprising 50 mRNAs, 49 methylated genes, 14 proteins, and 20 miRNAs. Subsequently, they separately applied hierarchical clustering using each type of the molecular data and successfully reproduced the two pancreatic cancer subtypes.

For graph-based neural networks, they take advantage of not only making use of the correlation among samples described by similar networks, but also message passing between targets and neighbors to improve the accuracy of targets identification^209^.

For example, to the best of our knowledge, the MOGONET proposed by Wang et al.^203^ is the first to make use of both graph convolution networks (GCNs) and cross-omics relationships in the label space for effective multiomics integration in biomedical data classification tasks. The specific process is as follows:

Firstly, they constructed a weighted sample similarity network for each type of omics data using cosine similarity. Taking both the omics features and the corresponding similarity network as the input, a GCN is trained for each type of omics data to predict class labels.

Secondly, the predictions generated by each omics data-specific GCN are further utilized to construct a new tensor, named cross-omics discovery tensor, which can reflect the cross-omics label correlations.

Finally, the cross-omics discovery tensor is forwarded to VCDN (view correlation discovery network) to explore the latent correlations across different omics data for final label prediction. Because the importance of a feature to the classification task can be measured by the performance decrease after removing individual features. Therefore, they used this method on the test data set to quantify and rank the contribution of each feature of different omics data to the prediction. Using the method, they identified top-ranking features as biomarkers for breast cancer.

In addition, Xuan et al.^204^ proposed a novel method based on the graph convolutional network and convolutional neural network (GCNLDA) to infer disease-related lncRNA candidates. First, they developed a network that is comprised of lncRNA, disease, and miRNA nodes. Then, they developed an embedding matrix of lncRNA-disease node pairs with respect to the biological premises. Then, they employed a convolutional neural network to explore various connections related to lncRNA-disease on node pair embedding. Finally, they learned the local network representations of lncRNA-disease pairs by deeply integrating the graph convolution autoencoder into topological lncRNA-disease-miRNA heterogeneous networks. Cross-validation confirmed that GCNLDA outperforms other state-of-the-art methods in terms of both AUC and AUPR^161^. Case studies^204^ on stomach cancer, osteosarcoma and lung cancer confirmed that GCNLDA effectively discovered potential lncRNA-disease associations. Therefore, GCNLDA is becoming an effective tool to screen reliable candidates for lncRNA-disease association validation with the help of biological experiments.

In summary, we consider that an increasing number of ML-based biology analysis applications will be developed to identify novel anticancer targets with the development of deep learning in the future.

### Evaluation of the druggability of potential targets

Druggability is a concept that assesses whether a drug can bind to a protein to alter its activity^3,4^. The human proteome has approximately 6,000 to 8,000 potential pharmacological targets, but only a small fraction can be targeted by drugs^7,210^. Therefore, it is important for us to evaluate druggability after finding novel anticancer targets. This study will introduce these applications from two perspectives: one is network-based biology analysis applications, and the other is ML-based biology analysis applications.

#### Network-based artificial intelligence for evaluating the druggability of potential targets

The druggability evaluating approach requires a long development cycle and high financial cost for the 3D structures of protein analysis^211^, while network-based biology analysis application provides an alternative methods to accelerate the evaluation procedure for the druggability of potential targets^212^.

**Fig. 10:**
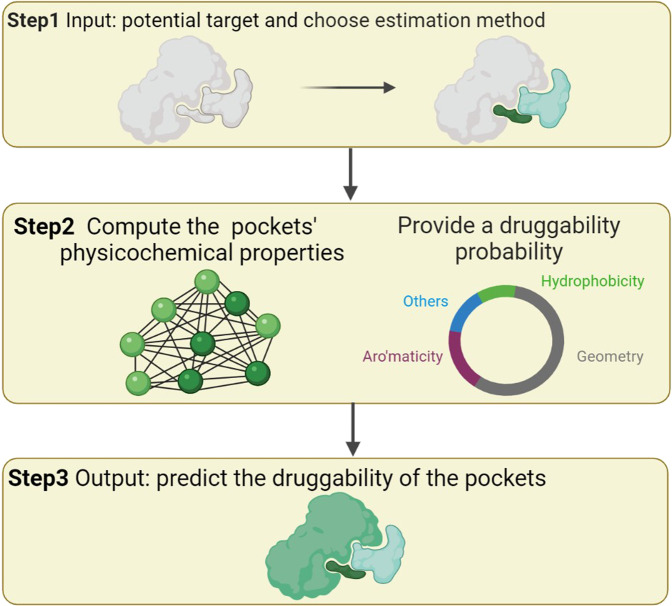
The workflow to evaluate the druggability of potential target proteins. (Created with BioRender.com)

Described by Fig. 10, PockDrug is a novel web server that is employed to predict pocket druggability on proteins and queried for a protein or a set of proteins^213^. For example, Yang et al.^214^ constructed a protein–protein interaction network for thyroid cancer and identified three key targets, HEY2, TNIK, and LRP4. Then, they used PockDrug to predict whether HEY2, TNIK, or LRP4 have targetable pockets for drugs in the following three steps.

In Step 1, they inputted the potential target and located pocket estimation methods. In Step 2, they predicted the druggability of the pockets by computing the physicochemical properties of the target pockets. In Step 3, they screened three hub genes, HEY2, TNIK, and LRP4. Based on the predictions, TNIK, which has 8 out of 538 residues, has an average druggability probability greater than 0.5 and thus was considered a druggable pocket for thyroid cancer.

In short, with the in-depth study of protein pocket, an increasing number of network-based biology analysis applications are developed to accurately evaluate the druggability of anticancer targets, providing reliable druggable targets for cancer treatment.

#### ML-based artificial intelligence for evaluating the druggability of potential targets

These ML-based biology analysis applications for evaluating the druggability of potential targets consist of protein structure modeling and drug-target affinity analysis. Previously, traditional analysis of protein structure modeling required considerable time and financial cost^211^, which greatly limited the traditional application of PockDrug since it is heavily dependent on an accurate 3D protein structure. Recent ML-based biology analysis applications have focused on developing methods to predict the 3D structure of a protein from its genetic sequence, also known as the protein folding problem. The cutting-edge ML-based modelling method^215–217^ can generate 3D protein structures with high accuracy and efficiency, which makes it possible for PockDrug to be widely used.

For example, Yang et al.^218^ developed the trRosetta algorithm, which fast and accurately predicts protein structures based on energy minimizations with restrained trRosetta. They employ a deep residual neural network to predict the restrained trRosetta, which consists of inter-residue distance and orientation distributions. Since trRosetta outperforms all previously protein modelling methods in benchmark tests on CASP13-^219^ and CAMEO-^220^ derived sets, it turns out that trRosetta can accurately predict protein structure. Furthermore, Senior et al.^221^ developed Alphafold to predict protein structures from amino acid sequences. First, Alphafold predicts the distances between pairs of residues by training a neural network to analyse the covariation of homologous sequences. Then, Alphafold constructs a potential mean force that accurately describes the shape of a protein. Finally, Alphafold optimizes the protein structure by a gradient descent algorithm. Because AlphaFold can predict protein structure with high accuracy even for such sequences with fewer homologous sequences, we consider that AlphaFold makes great progress in protein-structure prediction.

ML-based biology analysis applications for drug-target affinity (DTA) analysis application estimates the interaction strength of novel drug–target pairs based on previous studies to evaluate the druggability of targets^222^.

Compared with other methods, such as molecular docking^223^ and collaborative filtering^224^, graph-based neural networks are more effective in DTA prediction, because graph-based models facilitate the learning by considering both drug structure and drug-target interaction information instead of representing the drugs as string, as string sequences may lose the structural information of the molecule and may impair the predictive power of models^225^.

For example, Nguyen et al.^225^ is the first to use GNN for predicting DTA. The authors proposed GraphDTA, a new neural network model for regression tasks, which takes the drug-target pair as the input and outputs the continuous measurement of the binding affinity of the pair.

In detail, for the input drug-target pair, the protein targets are represented as sequence information instead of the molecular diagram of tertiary structure. While the drug compounds are represented as network graphs of atomic interaction, where each node is an eigenvector that represents five kinds of information: the atom symbol, the number of adjacent atoms, the number of adjacent hydrogens, the implicit value of the atom, and whether the atom is in an aromatic structure. For the output, GraphDTA combined the drug-target pair feature information to predict the continuous measurement of the binding affinity of the drug-target pair.

Through a multivariable statistical analysis of GraphDTA’s output data from hidden layers, the authors have two conclusions. One is to identify the correlations between hidden node activations and domain-specific drug annotations, such as the number of aliphatic hydroxyl groups, which suggests that the graph neural network can automatically assign importance to well-defined chemical features without any prior knowledge. The other is that the model makes it easier to extract features from drugs with obvious molecular structure patterns to achieve high-precision predictions. Especially, drugs that do not have an obvious molecular structure pattern are more difficult to predict.

In short, with the development of deep learning, an increasing number of ML-based biology analysis applications can quickly and accurately evaluate the druggability of anticancer targets, providing reliable druggable targets for cancer treatment and reducing the time and financial costs of experiments.

### Drug discovery

After evaluating the druggability of potential targets, it is essential to discover the drugs that interact with the potential therapeutic targets. As complex or concomitant diseases may usually require treatment with multiple drugs, but the use of multiple drugs will increase the risk of side effects^200^, it is very essential for drug discovery to predict the interactions between drug-target and drug-drug.

This study will introduce these applications from two perspectives as the above section: one is network-based biology analysis applications, and the other is ML-based biology analysis applications.

#### Network-based artificial intelligence for drug discovery

These network-based analysis applications for drug discovery consist of drug screening and drug repurposing. Drug screening is a process that potential drugs are identified and optimized before selecting a candidate drug to progress to clinical trials^226^. Since screening drugs through biological experiment is quite laborious, expensive, and time-consuming^226^, network-based biology analysis application becomes an alternative way for efficiently drugs screening.

Identifying drug-target interactions (DTIs) is crucial for drug screening. Especially, novel DTIs can be employed to look for the novel anticancer drugs with known targets^227^.

The network-based biology analysis applications for DTI prediction are usually based on guilt-by-association principle that a protein may be a target for a drug if many of the protein’s neighbors in the interaction network are targets of the drug^228^. Based on this principle, we classify the network-based biology analysis applications for predicting DTI into two categories.

One is ‘top-down’, which is from observable characteristics, such as side-effects or the diseases treated by a drug, to the interaction. For example, Campillos et al.^229^ used the physiological effect information from side effect similarity networks between entities for DTI prediction to predict whether two molecules could interact.

The other is ‘bottom-up’, which is from molecular features, such as protein structure, to interactions. For example, Feng et al.^230^ and Lee et al.^231^ predicted DTI based on the proteins in protein-protein interaction networks with similar property features that may interact with the same drug.

Drug repurposing, also known as drug repositioning, is another drug discovery application. It refers to a method that identifies new indications for approved drugs or drug candidates which have failed in the development phase^232^. Compared to the drug screening process, since drug repurposing can significantly reduce the drug development period and costs^233^, it is a better application to discover anticancer drugs.

The network-based biology analysis applications are efficient to carry out drug repurposing analysis, because the constructed drug similarity networks contain the similarity, interaction or linkages between drugs, diseases, and targets. Here, we introduce four major network-based biology analysis applications of drug repurposing^234–241^ as follows.

The first network-based biology analysis application of drug repurposing quantifies the similarities or relationships for known drug-disease associations, and then uses regression models or statistical models to predict novel drug-disease associations^234,235^. For example, Cheng et al.^242^ presented a network-based drug repurposing tool, which can accurately predicts drug responses in cancer cell lines by integrating human protein-protein interactome with transcriptome profiles, whole-exome sequencing, drug-target interactions and drug-induced microarray data.

The second network-based biology analysis application of drug repurposing infers new indications of drugs through analyzing information flow or performing random walks on drug-disease association networks^236–238^. For example, Luo et al.^243^ proposed a novel random walk method to measure the similarity of drugs and diseases respectively by the drugs properties and diseases properties, so as to predict potential indications of drugs.

The third network-based biology analysis application of drug repurposing, named individualized Network-based Co-Mutation, quantifies putative genetic interactions in cancer and it can be used to identify candidate therapeutic pathways for cancer^239^. For example, Cheng et al.^244^ used the approach to identify potential targets or new indications of existing cancer drugs that directly target significantly mutated genes or their neighbor genes in the human PPI interaction network.

The fourth network-based biology analysis application of drug repurposing can be realized directly through calculating the adjacency matrix of drug and disease network^240,241^. Based on this method, Luo et al.^245^ utilized the matrix completion algorithm to fills out the unknown entries in the drug–disease matrix by constructing a low-rank matrix approximation. New drug–disease associations will be screened by the predicted fill value.

Taken together, the network-based drug screening and repurposing applications provide researchers a lot of alternative approaches for quickly anticancer drugs discovery.

#### ML-based artificial intelligence for drug discovery

Currently, ML-based biology analysis applications have been employed to carry out drug screening and drug repurposing. For drug screening, previous studies have shown that network-based biology analysis applications can only screen the neighbour proteins of known targets, while drug-protein interactions may dysregulate the targets’ interacting neighbours^227^ resulting in high false positive prediction results. ML-based biology analysis applications, such as graph-based neural network, have the advantage of integrated features that combine both ‘bottom-up’^229^ and ‘top-down’^230^ approaches to reduce the high false positive prediction results.

**Fig. 11:**
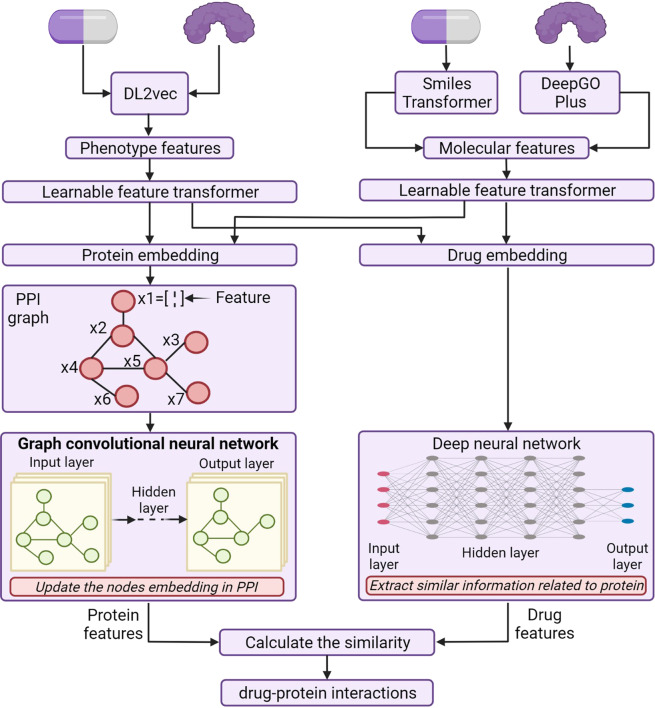
The graph-based neural network for DTI prediction by combining both bottom-up and top-down biology analysis approaches. (Created with BioRender.com)

For example, Hinnerichs et al.^227^ developed the DTI-Voodoo that combines molecular features and phenotypes information with an interaction network using graph neural networks to predict drug-protein interactions (Fig. 11).

Firstly, the model takes the two features, phenotypes features and molecular features, as input. To extracted phenotypes features, they utilized DL2Vec^246^ to obtain ontology-based representations. DL2vec constructs a PPI network by introducing nodes for each ontology class and edges for ontology axioms, followed by random walks starting from each node in the graph to generate representations that enable encoding drug effects or protein functions while preserving their semantic neighborhood within that graph. To extract molecular features, they utilized SmilesTransformer^247^ to capture the molecular organization of each drug from molecular structures of drugs and utilized DeepGOPlus^248^ to capture protein molecular features from protein amino acid sequences.

Secondly, they used two learnable feature transformer models to investigate the latent relationship between phenotypes features and molecular features. According to relationship information, the transformer model, which input the phenotypes features, will output the protein embedding for PPI networks (the top-down approach), and the other transformer model, which input the molecular features, will output drug embedding (the bottom-up approach).

Finally, a DNN was used to extract similar information related to protein from drug embedding, while a GCN is used to update the nodes embedding in PPI networks. Then both protein features and both drugs’ features are combined to calculate the similarity by cosine similarity. Since DTI-Voodoo performs well, it demonstrated that graph-based neural networks are good at identifying novel drug-protein interactions.

For drug repurposing, graph-based neural networks take the advantage of feature representation, which can not only utilize the drug-drug links information, but also the features between drug-cancer pairs.

For example, Cui et al.^249^ proposed GraphRepur, a model for drug repurposing prediction based on graph neural networks. Firstly, the authors collected the drug-induced gene expression data from the LINCS project^250^ as well as the drug-drug links information from the STITCH database^251^. Secondly, to obtain the signature of drugs, they identified differentially expressed genes for breast cancer and used the drug-induced genes from LINCS as drug signatures. Thirdly, based on the drug-drug links information from the STITCH database and drug signatures, they constructed a drug-drug links graph with drug signatures as node features. Fourthly, they input drug signatures and drug-drug links information into GraphRepur, and then the model computes scores for drugs that can be repurposed for treating breast cancer. Finally, the authors validated some predictive drugs for breast cancer using experimental data from the literature and showed that the model has significantly better performances than others, such as GCN, DNN, and random forest, in drug repurposing. using published studies.

Furthermore, the authors summarize three conclusions. The first conclusion is that the drug-drug links information plays an important role in studying drug repurposing. The second conclusion is that if such a network with fewer isolated nodes can provide a lot of network topology information, it will significantly improve the prediction performance of graph neural networks. The third is that the drug-induced genetic feature help to improve the DTI prediction accuracy of graph neural network.

Taken together, with the development of graph-based neural networks, an increasing number of ML-based drug screening and repurposing applications can quickly and accurately discover anticancer drugs, reducing the time and financial costs of experiments.

### Drug properties prediction

#### ADMET properties prediction

As discussed in section 4.3 (drug discovery step), after we have a list of drug molecules showing high affinity with the therapeutic target, it is necessary to investigate the properties of these candidates’ drugs^252–255^. Since the prediction of drug properties usually adopts the ML-based methods, this study mainly reviews the ML-based biology analysis applications for drug properties prediction such as the absorption, distribution, metabolism, excretion, and toxicity (ADMET) properties of chemical compounds^256^. Table 5 briefly described the ADMET properties.

**Table 5:** The brief description of the ADMET properties^256^

Property	Description
Absorption	The ability of a drug that cross membranes of many cell to reach its site of action, when drug is administered via oral ingestion.
Distribution	After absorption or systemic administration into the bloodstream, a drug is distributed to its site of action through the circulatory systems.
Metabolism	The process of chemically converting a drug to a metabolite is called metabolism or biotransformation.
Excretion	The collective term used for irreversibly removing a drug from the body
Toxicity	The extent to which a drug damages an entire organism, an organism’s substructure, or an organ.

**Fig. 12:**
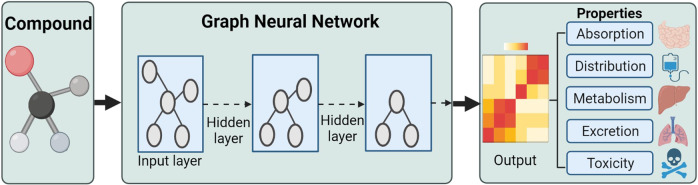
The graph-based neural network capture the features related to drug properties from drug molecular structure to predict ADMET properties of drugs. (Created with BioRender.com)

ADMET properties prediction can be considered as a classification or regression problem. Because of the strong ability of feature representation^177^, graph-based neural networks can capture the drug descriptors (the physicochemical properties, molecular representations, and drug-like properties of molecules) from the drug fingerprints (the substructure features of a molecule)^257^, so as to predict ADMET properties by classification or regression algorithm (Fig. 12)^258^.

For example, Duvenaud et al.^259^ proposed a graph convolution network to learn drug molecular fingerprints, which shows better performance than the state-of-the-art circular fingerprint method for ADMET properties prediction. After that, more and more scientists have used graph-based neural networks to predict the ADMET properties of drug molecules.

For example, Liu et al.^171^ proposed Chemi-Net, which utilizes GCN for ADMET properties prediction. They set the characterization of the atoms of the drug molecule and the relationship between atoms as the input of the Chemi-Net, while the output of Chemi-Net is the ADMET properties prediction of drug molecules. The predictive process of Chemi-Net is as follows.

Firstly, the model projects the assembling of the atoms and atom pair descriptors (features between atomic pairs)^257^ onto a 3D space to obtain a drug molecule-shaped graph structure. Secondly, Chemi-Net carries out a series of graph convolution operations to output a single fixed-sized molecule embedding. Finally, they obtain accurate ADMET properties predictions of drugs after passing the molecule embedding representation through fully connected layers.

In summary, we consider that more artificial intelligence models for drug properties prediction will be developed in the distant future.

#### The drug properties application in clinical trial

Since there have been a large number of applications based on artificial intelligence to study the properties of drugs, it still takes on average 10–15 years and 1.5–2.0 billion to bring a new drug to market^260^. One of the main stumbling blocks is the high failure rate of clinical trials. Therefore, some research are committed to the application of artificial intelligence for clinical trial design.

For example, Shah et al^261^ construct an artificial intelligence system that made use of the ‘self-learning’ deep reinforcement learning technology to looks at treatment regimens currently in use, and iteratively adjusts the doses. Therefore, the system can determine the fewest, smallest doses that could still shrink brain tumors, reduce toxicity and eventually find an optimal treatment plan with the lowest possible potency and frequency of doses that should still reduce tumor sizes to a degree comparable to that of traditional regimens. In simulated trials of 50 patients, the system designed treatment cycles that reduced the potency to less than a half of all the doses while maintaining the same tumor-shrinking potential.

In conclusion, we believe that with the development of artificial intelligence applications for drug property prediction, these applications will provide better help for clinical trial.

## Discussion and Conclusions

Modelling of cellular networks underlying cancer has provided us with a quantitative framework to investigate the link between network properties and the disease by artificial intelligence biology analysis, thereby leading to the discovery of potential novel anticancer targets and drugs^23–29^. However, there is no systematic review that introduces artificial intelligence biology analysis in cancer target identification and drug discovery. For this reason, this study briefly reviewed the scope of artificial intelligence biology analysis to explore new anticancer targets^34,54,57,74,80^, the principles and theory of commonly used artificial intelligence biology analysis algorithms^83–91^, and the artificial intelligence applications for artificial intelligence biology analysis^42,195,213^.

The scope of artificial intelligence analysis to explore novel anticancer targets consists of epigenetics^54^, genomics^57^, proteomics^74^, metabolomics^34^, etc. Since it is not accurate to have anticancer targets by single omics studies, we have to employ artificial intelligence biology analysis to effectively integrate multiple omics data and tackle the complexity of cancer that arises from interactions between genes and their products^16,17^ and improve our understanding of carcinogenesis^23–29^. Therefore, how to employ artificial intelligence biology analysis algorithms to integrate multiomics data and identify novel anticancer targets will be an important future study direction.

Next, we introduced two categories of commonly used artificial intelligence algorithms. One is network-based biology analysis algorithms and the other is ML-based biology analysis algorithms. We here discuss their limitations and advantages.

The network-based biology analysis algorithms usually are comprised of shortest path^83^, module detection^84^ and network centrality^85^, which have three major advantages: First, they provide a variety of alternative approaches to identify cancer targets, and different algorithms can compensate each other to identify targets from various perspectives, therefore providing new biological explanations^30^; Second, since they are not limited by the scale of the network, they are good at dealing with the case of small sample network; Third, prior biological knowledge and experience could be conveniently integrated into network-based biology analysis algorithms to make them interpretable.

However, previous studies also show two major shortcomings for the network-based algorithms: First, the current biological network data are biased toward much-studied targets^262^. Since previous studies have paid much attention to these targets, the network-based algorithms will more likely identify these well-studied targets than others due to the data bias^262^. Second, most algorithms only use the topological information of the biological network, but neglect the association between cell function or phenotypes and topological features (such as centrality-based algorithms that are discussed in Section 3.1.2).

ML-based biology analysis algorithms are usually comprised of decision trees^86–88^ and deep learning^89–91^, which have two major advantages.

One is feature learning and detection^177,181^, which employ sophisticated neural network architectures to link up features of biological networks and characterize their relationships. Subsequently, they iteratively train the model to detect such features that are hard to be detected by network-based biology analysis algorithms.

The other is their ability to effectively integrate large and diverse data. It is possible for ML-based networks biology analysis algorithms to integrate multiomics biological network data and identify novel targets^263^, because of the fast development of deep learning models and the easy access to high-throughput biological.

Although employing ML-based algorithms greatly benefits the target identification and drug discovery for cancer treatment^174^, we still have three major challenges to overcome.

The first challenge is the lack of consistent data for validation^33^. Although the recent advances in biotechnologies have enabled the fast generation of massive biomedical data, such data often suffer from inconsistency in production and information missing in annotation, resulting in the lack of reliable and consistent data for validating deep learning models^264^.

The second challenge is the integration of heterogeneous information^103^. Although deep learning models facilitate the integration of multimodal biological data, it is still difficult to build up a universal deep learning model due to the lack of biological domain knowledge^200^.

The third challenge is hard to provide interpretability of deep learning models^185^. However, a recent study sheds a light to resolve the issue through a combination of a disease network with a neural network to characterize the mechanism of melanoma^263^. In addition, graphs-based neural networks can improve the interpretability of deep learning models^265^.

In the last section of the study, we have reviewed the applications of artificial intelligence biology analysis for cancer therapy from four perspectives: novel anticancer targets identification^189^, evaluating the druggability of potential targets^3,4^, drug discovery^200^, and drug properties prediction^252–255^.

First, we presented several widely used applications to identify novel anticancer targets. However, exemplified by WGCNA^195^, these network-based biology analysis applications not only requires high computing costs to reconstruct gene co-expression networks^42^ but also has difficulty in accurately locating effective network nodes. Although ML-based biology analysis applications employ collaborative modelling by neighbourhood nodes information to reduce the computational cost and improve the predictive accuracy for anticancer targets, biological networks still have data bias^262^, resulting in most of the identified targets by current applications already have been reported in previous studies. Therefore, how to develop such an efficient feature selection application that can solve the data bias problem will be appealing for novel therapeutic anticancer target identification^266–268^ in the distant future.

Second, we introduce several widely used applications to evaluate the druggability of potential targets. For example, PockDrug is usually used to predict druggable pockets on proteins^213^. Although trRosetta^218^ and Alphafold^221^ offer opportunities for Pockdrug to evaluate the pharmaceuticals of potential targets, Pockdrug neither accurately predicts druggability due to the complexity of protein structure^269–271^ nor costs low efforts to validate through biological experiments^272,273^. Nevertheless, since DTA prediction can quickly provide reliable druggable targets for cancer care with low financial costs^211^, it is potential to develop the related efficient artificial intelligence biology analysis applications for DTA prediction in the distant future.

Third, we investigated several widely used applications for drug discovery, which consists of drug screening and drug repurposing.

For drug screening, identifying drug-target interactions (DTIs) is a crucial step. Since network-based biology analysis applications for DTI prediction are usually based on the guilt-by-association principle^228^, it can only predict the interacting neighbors of known cancer targets. Currently, ML-based biology analysis applications can extend the predictions to downstream consequences^227^, thereby screening out more possible anticancer drugs.

For drug repurposing^232^, there are four commonly used network-based biology analysis applications^234–241^ that integrate the similarities among various drugs but ignore prior knowledge. However, ML-based biology analysis applications not only can take advantage of the similarity among drugs, but also can integrate drug properties to improve the accuracy of drug repurposing.

Fourth, we introduce widely used applications for drug properties prediction. For example, graph convolution networks, which have a strong ability of feature representation^177^, can capture the features related to ADMET properties of drugs from their molecular structures. Therefore, it is becoming a popular method to predict drug properties by integrating drug molecular structures and drug clinical phenotype for drug properties prediction through graph convolution networks^274^. Here, we wish once more and more artificial intelligence biology analysis models are developed to capture the features related to ADMET properties from the drug molecular structure, to improve the success rate of clinical trials.

In summary, although we have reviewed and discussed many artificial intelligence algorithms and corresponding applications for novel anticancer target identification and drug discovery, this review is still too brief to cover the entire research area. However, because artificial intelligence algorithms are effective in exploring new anticancer targets and discovering drugs, we wish this review could offer valuable enlightenments for interested researchers to develop an understanding of the principles behind artificial intelligence biology analysis in cancer target identification and drug discovery. Moreover, we wish that our perspective on artificial intelligence and related applications will provide the pathway for further advancement in the field.
